# Ferroptosis-mediated immune responses in cancer

**DOI:** 10.3389/fimmu.2023.1188365

**Published:** 2023-05-30

**Authors:** Desheng Qi, Milin Peng

**Affiliations:** ^1^ Department of Emergency, Xiangya Hospital, Central South University, Changsha, Hunan, China; ^2^ National Clinical Research Center for Geriatric Disorders (Xiangya Hospital), Changsha, Hunan, China; ^3^ Department of Critical Care Medicine, Xiangya Hospital, Central South University, Changsha, Hunan, China

**Keywords:** cell death, ferroptosis, immunity, tumor microenvironment (TME), cancer immunotherapy

## Abstract

Cell death is a universal biological process in almost every physiological and pathological condition, including development, degeneration, inflammation, and cancer. In addition to apoptosis, increasing numbers of cell death types have been discovered in recent years. The biological significance of cell death has long been a subject of interest and exploration and meaningful discoveries continue to be made. Ferroptosis is a newfound form of programmed cell death and has been implicated intensively in various pathological conditions and cancer therapy. A few studies show that ferroptosis has the direct capacity to kill cancer cells and has a potential antitumor effect. As the rising role of immune cells function in the tumor microenvironment (TME), ferroptosis may have additional impact on the immune cells, though this remains unclear. In this study we focus on the ferroptosis molecular network and the ferroptosis-mediated immune response, mainly in the TME, and put forward novel insights and directions for cancer research in the near future.

## 1 Introduction

### 1.1 Discovery history of ferroptosis

The homeostasis of living entities is delicately regulated by the cell death of damaged cells and survival of properly working cells in a spatio-temporal manner. Cell death can take the form of either non-programmed cell death or programmed cell death (PCD). Necrosis is a non-programmed form of cell death that is induced by repeated freezing and thawing or other forms of physical tissue damage, and was identified as early as 1951 (1). Studies on cell death have increased in the past 50 years following the discovery of apoptosis (2). It has been found that apoptosis is closely involved in numerous biological conditions (3, 4), which is the first category of programmed cell death and is regulated by caspases. Previous researchers have generally considered apoptosis and necrosis to be the only cell death types of interest. However, this understanding has been challenged and many scientific discoveries in last two decades have brought to light new forms for cell death (5–8). Necroptosis is another fundamental programmed cell death and is found in the universal biological conditions of development, inflammation, and cancer (9–11), and pyroptosis is a Gasdermin D (GSDMD)-mediated PCD that is implicated in inflammation, immunity, and sepsis and other diseases (12).

**Figure 1:**
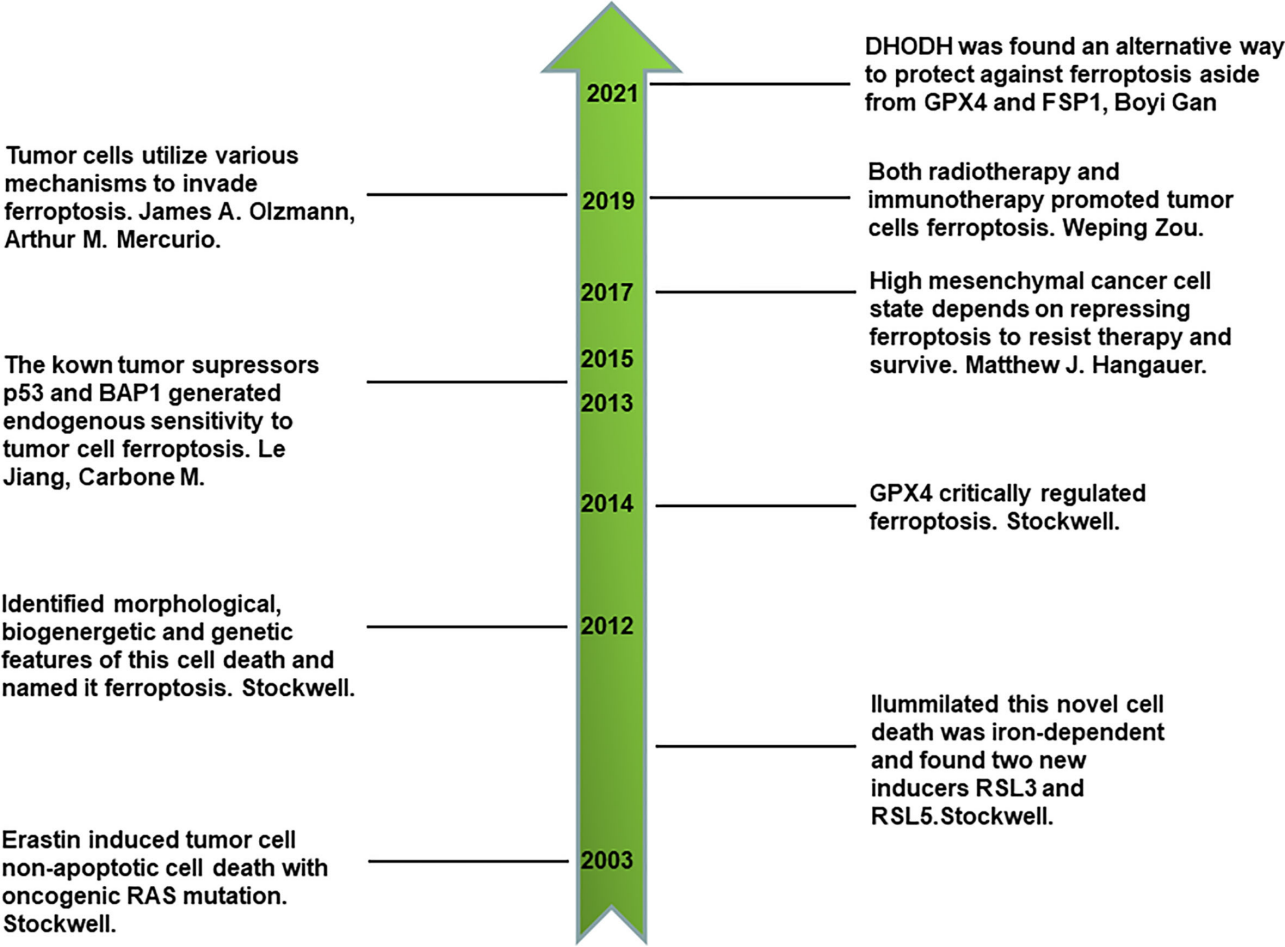
Milestones in the discovery history of ferroptosis.

Ferroptosis is a newly identified type of iron-dependent non-apoptotic cell death that was discovered in the last 10 years by Stockwell (7, 13, 14). By reviewing its discovery history, we can obtain a comprehensive understanding of ferroptosis. First, a novel compound, erastin, was identified to selectively kill engineered cancer cells expressing oncogenic RAS and small T oncoprotein in a non-apoptotic manner through compounds screening for engineered human tumor cells (13). This first discovery led to much more comprehensive studies. Interestingly, using the synthetic lethal screening modality *in vitro*, Stockwell found that the introduction of oncogenic RAS into cancer cells leads to increased levels of cellular labile iron pool by upregulating transferrin receptor gene TfR1 and downregulating ferritin genes *FTH* and *FTL*. This non-apoptotic cell death is inhibited by iron chelator, which indicates that it is iron-dependent. In addition, two other compounds, RSL3 and RSL5, were identified as inducers of this novel form of cell death. RSL5 and erastin both lead to this cell death by targeting voltage-dependent anion channel 3 (VDAC3), whereas RSL3 induces death in a VDAC3*-*independent manner (14). VDACs are mitochondrial pores that transport small metabolites and ions. Furthermore, ferroptosis is named to represent this iron-dependent non-apoptotic cell death after one more key study (7), in which ferroptosis was proven to have unique morphological, bioenergetic, and genetic traits that are distinct from previous identified types of cell death. Cells undergoing ferroptosis have the morphological features of small and shrunken mitochondria under electron microscope scan. In terms of bioenergetics, ferroptosis does not consume much intracellular adenosine triphosphate (ATP) nor rely on mitochondrial electron transport chain (ETC) to generate reactive oxygen species (ROS). Lipid peroxidation was found to be another characteristic of ferroptosis. As for genetic features, six high-confidence genes were found to significantly change in cells undergoing ferroptosis. These were *IREB2* (iron response element binding protein 2), *RPL8* (ribosomal protein L8), *CS* (citrate synthase), *ATP5G3* (ATP synthase F0 complex subunit C3), *ACSF2* (acyl-CoA synthetase family member 2), and TTC35 (tetratricopeptide repeat domain 35) (7). There are other landmarks in the history of ferroptosis. Glutathione peroxidases 4 (GPX4), a key component of keeping intracellular reducing state, plays a crucial role in the regulation of ferroptosis (15). P53, a canonical tumor suppressor, sensitizes ferroptotic cancer cell death (16). BAP1, another tumor suppressor, also sensitizes ferroptotic tumor cell death by repressing the amino acid antiporter system Xc^-^ expression, which is important for synthesizing cellular reduced glutathione (GSH) to protect lipids from peroxidation and ferroptosis (17). Thus, ferroptosis is closely associated with conventional tumor suppressors’ activities and is an endogenous mechanism that represses the survival of tumor cells. Inversely, tumor cells utilize various mechanisms to evade ferroptosis, such as upregulating ferroptosis suppressor protein 1 (FSP1) (18). In addition, prominin 2 promotes ferritin-containing exosomes to transport iron out of cells and protects cancer cells from ferroptosis stress (19). Primary tumor growth relies on the inhibition of endogenous ferroptosis. Drug-resistant and highly mesenchymal cancer cells depend on GPX4 activity to repress ferroptosis and survive (20, 21). Furthermore, immunotherapy-activated CD8^+^ T cells induces the ferroptosis of tumor cells by secreting interferon gamma (IFN-γ) and inhibiting system Xc^-^ on the cell membrane (22). Radiotherapy also directly induces the ferroptosis of tumor cells, which is also a novel junction of synergy between radiotherapy and immunotherapy (23). Recently, it was also found that dihydroorotate dehydrogenase (DHODH) is an alternative way to protect against ferroptosis aside from GPX4 and FSP1 (24). All the above milestones comprise a sketch of the discovery history of ferroptosis (**Figure 1**).

## 2 Molecular network of ferroptosis

### 2.1 General portrayal of ferroptosis molecular network

**Figure 2:**
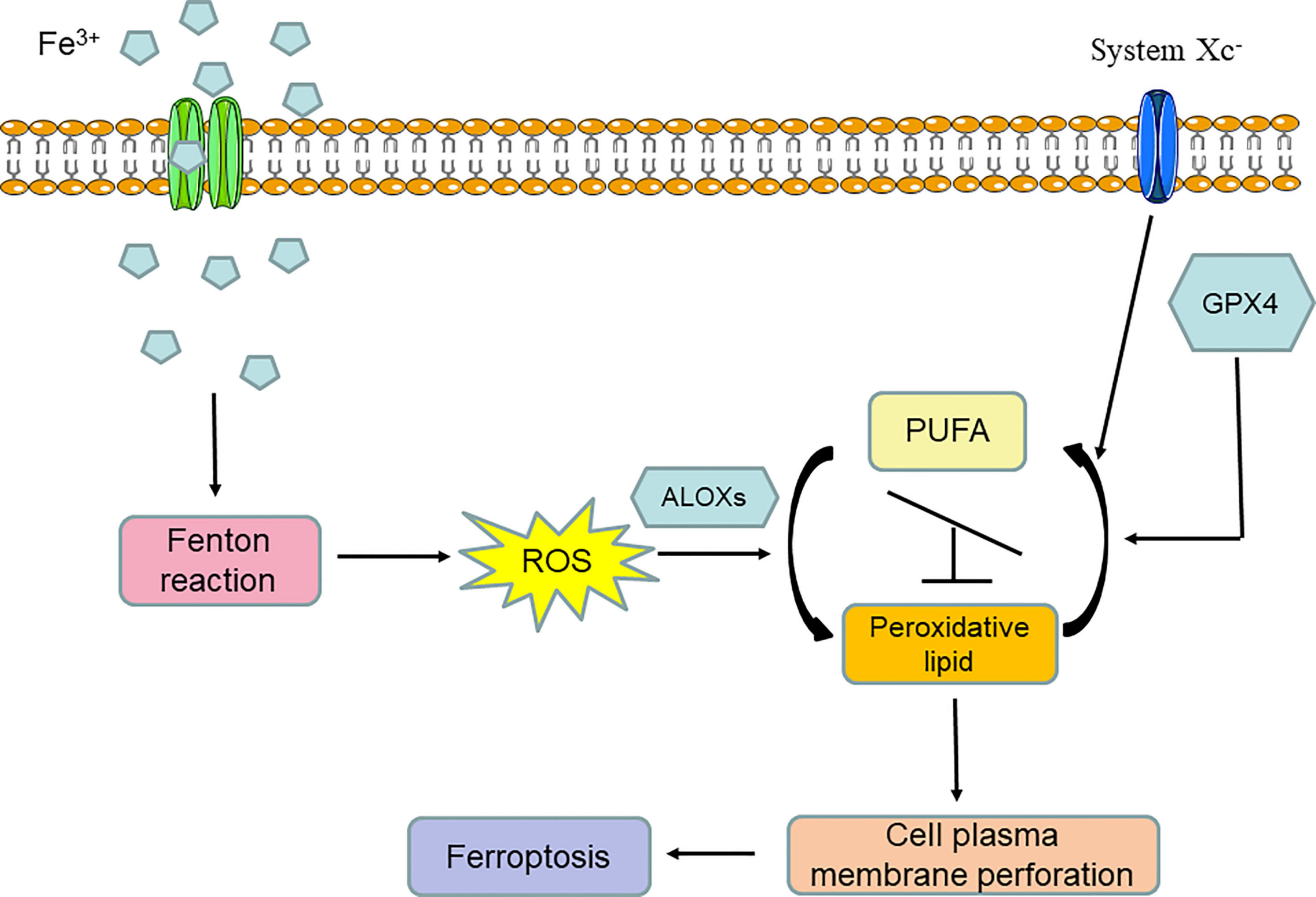
Molecular network of ferroptosis.

From the above introduction to the discovery history of ferroptosis, we can conclude that ferroptosis is an iron-dependent programmed form of cell death that is induced by lipid peroxidation and the breaking of the intracellular reducing state maintained by amino acid and antioxidation metabolism. Thus, ferroptosis is by nature a form of metabolic cell death mainly involved in iron metabolism, lipid metabolism, and amino acid and antioxidation metabolism. In general, iron overload can induce the Fenton reaction and lead to the significant accumulation of oxygen free radicals in cells. When the oxidizability induced by oxygen free radical overpowers the reducing state that is mainly maintained by GPX4 and system Xc^-^ activity, arachidonate lipoxygenases (ALOXs) are activated and turn polyunsaturated fatty acids (PUFAs) into peroxidative lipids; this is known as lipid peroxidation. Lipid peroxidation can lead to cell plasma membrane remodeling and perforation, after which cell normal structure is destroyed. Finally, ferroptosis occurs (**Figure 2**).

### 2.2 Iron metabolism

As shown above, iron overload is the critical initial procedure for ferroptosis, and iron metabolism is heavily involved in the cancer microenvironment. Malignant cells rely on high levels of intracellular iron to fulfill the biosynthesis of DNA and proliferation (25). However, accumulated iron in tumor cells is a double-edged sword, as excessive labile iron in cytoplasm can also elicit the Fenton reaction, which induces lipid peroxidation and lipid toxicity (26). Iron-dependent lipid peroxidation can generate an overload in the levels of oxygen free radicals, which eventually leads to ferroptosis. A number of genes and proteins implicated in iron metabolism have been reported to regulate ferroptosis. When inducing ferroptosis, the iron-starvation response is activated and results in the downregulation of ferritin (genes: *FTH1* and *FTL*; function: store intracellular iron) and ferroportin (function: export iron out of the cell), and the upregulation of transferrin receptor (gene: TfR1; function: import iron into the cell) (14), which leads to the accumulation of intracellular free iron pool. The iron–sulfur cluster biosynthetic enzyme NFS1 maintains the iron–sulfur co-factors level and serves as an upstream regulator to control the iron-starvation response and limit the effect of high levels of oxygen tension in a lung adenocarcinoma context. NFS1 suppression sensitizes lung adenocarcinoma cells to ferroptosis by stimulating the iron-starvation response, stabilizing TfR1 mRNA and inhibiting FTH1 expression (27). Another critical mediator is nuclear receptor coactivator 4 (NCOA4), which is required for transporting ferritin to lysosomes to degrade, resulting in the release of ferritin-bound iron to increase intracellular iron levels (28). Heme oxygenase-1 (HO-1), a main source of intracellular iron, has been proven to play a decisive role in erastin-induced ferroptosis, and the inhibition of HO-1 prevents erastin-triggered ferroptosis in HT-1080 fibrosarcoma cells (29). However, in hepatocellular carcinoma cells (HCCs), the knockdown of HO-1 by small interfering RNA facilitates ferroptosis induced by sorafenib and erastin (30). Thus, the role of HO-1 in ferroptosis is still in debate and is specific to different cells. In addition, the p62-Keap1-nuclear factor erythroid 2-related factor 2 (NRF2) pathway serves as an upstream modulator regulating HO-1 and FTH1 stability and protecting HCCs from ferroptosis (30). Fanconi anemia complementation group D2 (FANCD2) also protects bone marrow stromal cells from ferroptosis by regulating the genes or proteins in iron metabolism (31). Divalent metal transporter 1 (DMT1) is another key regulator involved in iron metabolism and it has four isoforms. DMT1A1, one of the isoforms, is localized on the apical cell membrane and has the function of non-transferritin-bound ferrous iron uptake. DMT1A/B-II, localized at the endosomal membrane, imports ferrous iron from endosome to cytosol (32). Importantly, human poly (rC)–binding protein 1 (PCBP1) acts as an iron chaperone that delivers ferrous iron to ferritin for storage in the cytoplasm (33), which can protect cells from labile ferrous iron-related oxidative damage. However, the relationship between DMT1, PCBPs, and ferroptosis has not yet been explored and needs further study. Phosphorylase kinase G2 (PHKG2) is another mediator of cellular labile iron pool tha facilitates ferroptosis (34). In a word, these markers in iron metabolism probably have a close relationship with ferroptosis and can be utilized for further investigation.

### 2.3 Lipid metabolism

Iron-associated Fenton reaction is the catalyzer for ALOXs activity, resulting in lipid peroxidation and ferroptosis. In addition, PUFAs are the specific substrates for this biochemical reaction. The bis-allylic position in PUFAs is the critical site for the production of peroxidation and determines the radical chain reaction of lipid peroxidation (34). Acyl-CoA synthetase long chain family member 4 (ACSL4) and lyso-phosphatidylcholine acyltransferase (LPCAT) play important roles in the esterification of PUFAs into membrane phospholipids, which are the key signals for the stimulation of ferroptosis (35, 36). PUFAs insert into cell membrane phospholipids and formulate PUFAs-containing membrane phospholipids, such as phosphatidylethanolamines (PEs) containing arachidonic acid (C20:4) and adrenic acid (C22:4) (36). Recently, polyunsaturated ether phospholipid (PUFA-ePL) was found to be another important substrate for lipid peroxidation reaction, eventually leading to ferroptosis (37). PUFAs-containing PE phospholipids on cell membranes are oxidated by the ALOXs family (34, 36, 38). There are six ALOX genes in the human ALOXs family: *ALOXE3*, *ALOX5*, *ALOX12*, *ALOX12B*, *ALOX15*, and *ALOX15B*. These six genes have different expression levels in various cell lines and tissues. The human renal carcinoma cell line G-401 expresses all six ALOX genes. The silencing of all six ALOX genes drives G-401 cell resistance to ferroptosis inducer imidazole ketone erastin (IKE) treatment rather than (1S, 3R)-RSL3 stimulation (34). IKE inhibits system Xc- on cell membrane to exhaust cellular GSH and inhibits GPX4 indirectly (like erastin function), whereas (1S, 3R)-RSL3 suppresses GPX4 activity directly in the absence of cellular GSH exhaustion (39). BJeLR or HT-1080 cell line does not express the *ALOX5*, *ALOX12*, *ALOX12B*, or *ALOX15* genes but does express *ALOXE3* and *ALOX15B*. The silencing of *ALOXE3* and *ALOX15B* in the BJeLR or HT-1080 cell lines rescues erastin-induced ferroptosis, which also confirms that ferroptosis that occurs after GSH exhaustion requires ALOXs (34). In addition, *ALOX12* and *ALOX15* are specific downstream markers of ferroptosis that work by activating apoptosis-inducing factor (AIF). Cell death is completely prevented by inhibiting *ALOX12* and *ALOX15*, or silencing AIF (38). The detailed role of AIF in ferroptosis needs further study. Supplement of PUFAs together with transforming growth factor-β1 (TGF-β1) synergistically inhibit B16 cell growth, and this effect can be completely reversed by the antioxidant vitamin E, but adding PUFAs alone does not inhibit B16 cell growth (40). As vitamin E is a potential anti-ferroptosis agent (41, 42), further study is needed to confirm whether TGF-β1-mediated B16 cell growth inhibition and enhancement by PUFAs is due to the inducement of ferroptosis. In addition, mevalonate metabolism is implicated in ferroptosis pathway (43). However, the total net effect of mevalonate pathway on ferroptosis is still undetermined and requires further study (43, 44).

### 2.4 Amino acid and antioxidation metabolism

The intracellular reducing state is an aspect of homeostasis that is crucial to the maintenance of normal function in living beings. Superoxide anion radical (O2^•–^) and hydrogen peroxide (H_2_O_2_) are the two main types of reactive oxygen species (ROS) (45). Oxidized lipid is another important kind of ROS. The influences of ROS on inflammation, cancer, senescence, and other pathologic conditions are prevailing and vital (46–48). In cells, there are certain mechanisms that regulate ROS levels. Amino acid metabolism, especially that of cystine and glutathione, has the key function of controlling ROS and maintaining an antioxidant state in normal cells. The cystine and glutathione metabolic pathway under normal circumstances is as follows. System xc^–^ on the cell membrane is made up of the disulfide-linked heterodimers SLC3A2 and xCT and serves as the bidirectional channel to transfer cystine into cells and export intracellular glutamate out at a 1:1 ratio. Subsequently, intracellular cystine and glutamate are synthesized into GSH by glutamate-cysteine ligase (GCL) and glutathione synthetase (GSS). Some cells take advantage of the transsulfuration pathway to produce cysteine from methionine, thus bypassing the system xc^–^ function of importing cysteine. GSH is the cofactor of GPX4 in its reduction of phospholipid hydroperoxides to phospholipids, which is the unique function of GPX4 (49). GPX4, a member of the glutathione peroxidases family (GPXs), is the core mediator to maintaining an antioxidant state in cells. The disruption in the above antioxidant mechanism by inhibiting key targets such as system xc^–^, GCL, and GPX4 generates the accumulation of ROS and lipid peroxides, leading to ferroptosis (7, 15). GPX4 is the key regulator in the ferroptosis pathway (15). By using 60 different human cells in the NCI-60 cell line panel, it was found that intracellular nicotinamide adenine dinucleotide phosphate (NADPH) abundance is negatively correlated with ferroptosis sensitivity (50). All ROS are generated by mitochondrial electron transport chain (ETC) complexes and NADPH oxidases. While in ferroptosis, cells eliminated from the mitochondria still retain their sensitivity to ferroptosis, indicating that ROS generated by mitochondria are dispensable for inducing ferroptosis (7). However, inhibiting ETC complexes in mitochondria blocks ferroptosis induced by system xc^–^ inhibition, but not by GPX4 suppression (51). The true underlying mechanism remains obscure, and the role of mitochondria in ferroptosis is still undetermined and, furthermore, more comprehensive studies are needed. In addition, the inhibition of thioredoxin reductase (TXNRD) activity induces ferroptosis through upregulating Ptgs2 expression (52). In a word, amino acid and antioxidant metabolism, and associated key regulators, are closely related to ferroptosis.

### 2.5 Glucose metabolism and regulation of ferroptosis by cancer suppressor gene

Glucose metabolism has a close correlation with ferroptosis. The tricarboxylic acid (TCA) cycle, the fundamental form of glucose metabolism, has close links with ferroptosis. The mitochondrial TCA cycle and ETC complexes promote cysteine-deprivation-induced (CDI) ferroptosis by inducing oxidative phosphorylation (OXPHOS) and producing cellular lipid peroxides (51). Glutaminolysis is also deeply implicated in the regulation of ferroptosis through its replenishing of TCA cycle intermediates such as α-ketoglutaric acid (α-KG). Glutamine is absorbed in cells by SLC1A5 transporters on the cell membrane. Subsequently, glutamine is transformed to glutamate by glutaminase (GLS1) in the cytoplasm. Glutamine can also enter mitochondria and form glutamate by glutaminase (GLS2). Glutamate in mitochondria can be turned into and subsequently supply α-ketoglutaric acid (α-KG) for TCA cycle through its reaction with glutamic-oxaloacetic transaminase 1 (GOT1). Suppressing glutamine uptake by the SLC1A5 transporter, or inhibiting glutamine transformation to glutamate by GLS2, or restraining glutamate conversion to a-KG in mitochondria, all inhibit ferroptosis (53). In addition, as we know, fumarate hydratase (FH) is an enzyme in the TCA cycle that catalyzes the transformation of fumarate into malate and has the function of suppressing cancer and the DNA damage response pathway (DDR). Intriguingly, *FH-*inactivated renal cell carcinoma (HLRCC) exhibits oxidative stress promotion, and metabolic alteration, and increased sensitivity to ferroptosis through the mechanisms of inducing fumarate accumulation, the succination of GPX4 proteins at the C94 site, and the inhibition of GPX4 function (54). On the other hand, *FH-*inactivated HLRCC also demonstrates the fumarate accumulation-related attenuation of NRF2 degradation (54), which is the notable protective factor in the inhibition of ferroptosis (30, 55, 56). The net effect of ferroptosis on *FH-*inactivated HLRCC is still unknown, and further, more comprehensive studies are needed to determine the impact of fumarate hydratase on ferroptosis in other cancers. This entry point is interesting and reflects the in-depth correlation between glycometabolism and ferroptosis through post-translation modulation in the form of succination, which is a promising future research direction. In addition to, *FH* as cancer suppressor gene involved in cancer cell ferroptosis, two other cancer suppressor genes, *p53* and *BAP1*, have the direct function of repressing the expression of system xc^-^, inhibiting cystine uptake, and sensitizing cancer cells to ferroptosis (16, 57, 58). In a word, cancer suppressor genes utilize ferroptosis, by means of glycometabolism modification, as a critical endogenous way of inhibiting tumor growth.

## 3 Ferroptosis’ role in tumor immune microenvironment

Ferroptosis has not only a complex molecular network but also vital functions in many pathologic conditions, including acute kidney injury, cardiomyopathy, traumatic nerve injury, and neurodegenerative diseases (59–62). In addition, as ferroptosis was firstly discovered using high-throughput compounds screening for killing tumor cells (13), it has been intensely studied by those in the tumor research field over the years. Some intractable cancer cells that are resistant to apoptosis, necroptosis, pyroptosis, and other cell death types, have been found to be susceptible to ferroptosis. A therapy-resistant state, depending on the lipid peroxidase pathway, can be induced in cancer cells by ferroptosis (21), meaning that ferroptosis is potentially a viable way to cure cancer. This topic can be dissected into two main aspects; one is the effect of ferroptosis on tumor cells themselves, and the other is the role of ferroptosis in the tumor microenvironment (TME). The first aspect has been continuously explored since the discovery of ferroptosis. In subcutaneous xenograft mouse tumor models, Stockwell found that the ferroptosis inducer (1S, 3R)-RSL3 can suppress tumor growth in athymic nude mice (15). Another study showed that IKE, a ferroptosis inducer, exerted an antitumor effect in an immunodeficient mouse lymphoma model (63). However, recent research has demonstrated that the compound of ferroptocide can induce ferroptosis in an immunocompetent murine model of breast cancer, but not in immunodeficient mice (64). Zou reported that cyst(e)inase alone, an ferroptosis inductive agent, could not suppress ovarian cancer ID8 growth, but that the combination of cyst(e)inase and anti-PD-L1 could inhibit ID8 development (22). These studies indicate that ferroptosis has the direct effect of killing tumors and also of activating an antitumor immune microenvironment. On the other hand, many kinds of tumor cells are equipped with various defense mechanisms to withstand ferroptosis, such as transporting iron out of cells by way of exosomes, or halting lipid peroxidation by way of ferroptosis suppressor protein 1 (FSP1) (18, 19). However, the metabolic vulnerability of tumors provides therapy opportunities (65), and drug-tolerant persistent cancer cells rely on GPX4 activity to resist treatment (20). Thus, inhibiting GPX4 to induce ferroptosis is considered to be a useful means of taking advantage of this “Achilles heel” to inhibit cancer progression. Although there is increasing interest in utilizing ferroptosis to kill tumor cells directly and suppress tumor growth, the role of ferroptosis in the TME remains obscure. In consideration of the pivotal role of TME in cancer progression, the aim of this review is to investigate the potential impact of ferroptosis on TME, in order to highlight a crucial and valuable field for future investigation.

### 3.1 Ferroptosis associated innate immune response

The role of ferroptosis in the TME was first explored in cell death-related immunological studies. From the first and second Cold Spring Harbor Symposium and the notable work of immunologist CA Janeway, Jr., a more comprehensive and profound conception of immunity from an evolutionary perspective was put forward, in particular Burnet’s crucial clonal selection theory, which posited that the first signal of class I major histocompatibility complex molecules (MHC-I) was their interaction with T-cell receptors, that the second signal was a co-stimulator to activate T cells, and that the initial signal of pattern recognition receptors (PRRs) to recognize pathogen-associated molecular patterns (PAMPs) and damage-associated molecular patterns (DAMPs) by antigen-presenting cells (APCs) was also vitally important for immune system function (66). Cross-priming is the prevailing and critical model used by APCs, mainly dendritic cells (DCs), to process extrinsic protein antigens and present to T cells through MHC-I (67). The self/non-self model of immunity comprises the microbial non-self, missing self, and altered self (68). The microbial non-self contains extrinsic invading pathogens, and in the missing self contains cells with decreased expressions of MHC-1, CD47, or sialic acid, which are “do not eat me” signals. Dying cells belong to the altered self and release DAMPs, which are “find me” and “eat me” signals that either stimulate or suppress immune responses. In addition, there is an emerging theory in the field of cancer research regarding the association of dying cancer cells with immune responses through their mimicry of pathogen defense responses. Immunogenic dying cancer cells exhibit behavior that is similar to pathogen-infected dying cells, on the one hand releasing chemokines such as CXCL1, CCL2, and CXCL10 (pathogen response-like chemokine signature, PARC) that recruit neutrophils as first responders to phagocytose dying cells. On the other hand, dying cancer cells release DAMPs, including ATP, nucleic acids that can be recognized by APCs, activates neutrophils and eventually facilitates nitric oxide-based respiratory burst and hydrogen peroxide release, which causes the death of residual viable cancer cells (68, 69). In this way, the altered self-mimicry of dying cells can be perceived by the innate immune cells to clear residual dying cells. Numerous clinical studies have shown that T cell-infiltrated tumors respond sensitively to immunotherapy (70, 71), whereas in non-T cell-infiltrated tumors, other means of maximally expediting innate immune activation in the TME are required (72). Immunogenic cell death (ICD) was found to have an antitumor capacity by activating immune response (73–76) and is an emerging determinant in various clinical cancer therapeutic modalities, including radiation therapy, chemotherapy, molecular targeted therapy, and immunotherapy (77–81). Whether or not ferroptosis has an immunoregulatory effect in the TME is still being explored. The immune system is composed of two main parts: innate immunity and adaptive immunity. The components of innate immunity consist of physical/chemical barriers, humoral innate immunity, and inherent immune cells (82). External protective structures, external mucous secretions, and specialized skin and mucosa form physical/chemical barriers. Lysozymes, antimicrobial peptides, acute phase proteins, complements, cytokines, and natural antibodies make up humoral innate immunity. Intraepithelial T lymphocytes, myeloid phagocytic cells, innate lymphoid cells, phagocytic B cells, non-specific cytotoxic cells, and mucosa-associated invariant T cells are types of inherent immune cells (82). Innate immunity works as the first guard to kill invading pathogens and mutant cancer cells by phagocytosis, extracellular traps, and other mechanisms, and is therefore critical to the maintenance of homeostasis. The innate immune system is also contains sentry cells that recognize the “not me” signal, and processing and presenting the alien antigens to adaptive immune cells. Efforts have been made to explore the effect of ferroptosis on innate immunity. Keratinocytes, the protective shelters of skin, show features of cell death including ferroptosis *in vitro* and *in vivo* by specifically knocking out the glutamate-cysteine ligase gene in keratinocytes (83). Macrophages are the main drivers of innate immunity. An *in vitro* study shows that when human leukemic Jurkat T cells were induced to ferroptosis and then co-cultured with macrophages, this led to the exposure of phosphatidylserine being detected on the surface of dying cells, and enhanced phagocytosis by macrophages (84). Ferroptosis can promote the release of KRAS^G12D^ protein from pancreatic ductal adenocarcinoma cells (PDAC). KRAS^G12D^ protein is then delivered to macrophages by exosomes and stimulates macrophage polarization to the M2 pro-tumor type (85). A recent study discovered that proteoglycan decorin (DCN), as the novel DAMP released by cells dying from ferroptosis, can trigger the production of inflammatory macrophage cytokines and elicit an anticancer immune response (86). In addition, the knockdown of MXRA8 can elicit ferroptosis in glioma cells and decrease the infiltration of M2 macrophages (87). One study indirectly proved that the inhibition of ferroptosis might prevent macrophage polarization to the M1 pro-inflammatory type and limit the release of TNF-*α* and IL-1*β* in folic acid-induced kidney injury conditions (88). In addition, one study found that M1 type macrophages exert high resistance to pharmacologically induced ferroptosis, whereas M2 type macrophages are sensitive to ferroptosis in TME conditions and brain trauma under regulation by inducible nitric oxide synthase (iNOS)/NO^•^ (89). In general, ferroptosis may result in enhanced phagocytosis and clearance abilities of macrophages, and the increase of the M1/M2 type macrophage ratio in some conditions, which is a desirable outcome for improving the TME packed with immunosuppressive M2 type macrophages.

As for other types of innate immune cells, one study demonstrates that photosensor photodynamic therapy (PS-PDT) induced the immunogenic cell death of glioma GL261 and fibrosarcoma MCA205 cells. This process was inhibited by ferrostatin-1 and DFO (ferroptosis inhibitors), which indicated that ferroptosis was involved in PS-PDT-induced cell death. After co-culturing, PS-PDT-induced dying cells were more effectively engulfed by bone marrow-derived DCs (BMDCs) than live cells, and BMDCs were efficiently engulfed by BMDCs. And BMDCs were then matured and activated of surface co-stimulatory molecule CD86, MHCII expression, and releasing higher interleukin-6 (IL-6) (90). The results indicate that cells undergoing ferroptosis promote the production of BMDCs to gain a higher phagocytotic capacity, maturation, and activation, and that ferroptosis is a type of ICD. Another study found that ferroptosis was the cell death type for cells in an injured myocardium, and orchestrated the recruitment of neutrophils to the injured myocardium after heart transplantation through a toll-like receptor 4 (TLR4)/TIR-domain-containing adapter-inducing interferon-β (TRIF)/type I interferon (IFN) signaling pathway (91).

When discussing the interaction between cancer cells and innate immune cells in the TME in depth, there is a series of steps that we followed in our attempt to shed light on this process. The first step is how immune cells distinguish altered self ferroptosis cancer cells from normal self cells in the early stages before rupture. We hypothesize that cell membrane epitopes on the outside surfaces of tumor cells are reprogramming when undergoing ferroptosis and serve as “find me” and “eat me” signals. What is the initial signal in the interaction between ferroptosis cells and innate immune cells? In a study comparing three types of cell death, that is apoptosis, necroptosis, and ferroptosis, Katharina detected phosphatidylserine exposure on the outside membranes of all three types of dying cells, subsequently recognized and engulfed by macrophages *in vitro* (84). As phosphatidylserine exposure is the notable characteristic of apoptotic cells to be recognized by macrophages and induces the clearance of residual apoptotic cells and immune tolerance, the subsequent effect of phosphatidylserine exposure on ferroptosis cells in the TME remains unclear. In another study using the dynamic lipid membranes model, Stockwell simulated ferroptosis cell membrane by combining a high concentration of PUFAs, lipid peroxides, and longer acyl tails, and found that a higher composition of oxidized phosphatidylethanolamine (PE-AA-OOH) on the membrane was the main characteristic of cell membrane change in ferroptosis cells, which was probably the main cause of ferroptosis cell membrane rupture and final death (92). Another study found that the oxidized phospholipid of 1-steaoryl-2-15-HpETE-sn-glycero-3-phosphatidylethanolamine (SAPE-OOH) was a key “eat me” signal on the ferroptosis cell surface that could be recognized by TLR2 on macrophages (93). The oxidation of arachidonic acid via ACSL4, acyl-CoA:lysophosphatidylcholine acyltransferase (LPCAT), and ALOX enzymes generated PE-AA-OOH production. Other types of eicosanoids are also produced in the context of ferroptosis, including prostaglandin E2 (PGE2), oxidized phospholipids (oxPLs), 5-Hydroxyeicosatetraenoic (5-HETE), 11-HETE, 12-HETE, and 15-HETE (94). Multiple studies have extensively explored the crucial immunometabolic modulatory function of eicosanoids interaction with inflammatory cells by specific receptors (95). However, in the TME, a selective inhibitor of 20-HETE synthesis, HET0016, was reported to be able to decrease breast tumor volume and lung metastasis and reduce the population of granulocytic myeloid-derived suppressive cells (MDSCs) (g-MDSCs: CD11b^+^Ly6G^+^) and the expression of pro-inflammatory cytokines (96). As it has been established that MDSCs are capable of constructing an immunosuppressive niche in favor of tumor growth, it was expected that 20-HETE would have the ability. Furthermore, we learn from previous studies that the immunometabolic modulatory function of eicosanoids has a specificity that is time- and space-dependent (97). The evolution process of specific types of eicosanoids on the surface of ferroptosis cells, varying with time and space, may exert diverse effects on associated immune responses in tumor immune niche in different stages of ferroptosis, and this needs future study. Except for cell membrane lipid metabolism reprogramming on the surface of ferroptosis cells determining immune response, the second step of interaction between ferroptosis cells and immune cells may closely correlate to DAMPs, which are endogenous messages that deliver signals to immune cells or non-immune cells by binding specific receptors. It has been reported that certain DAMPs have the capacity to trigger the recruitment of monocytes and neutrophils to activate inflammatory responses (98) and serve as the initial signal to induce autophagy and immunity (99). The release of DAMPs is the hallmark of ICD. The immunogenicity of DAMPs, as “find me” or “eat me” signals that emitted from dying cells, determines their ability to activate immune responses, which has a beneficial function in antitumor therapy (100). The common types of DAMPs include high mobility group protein B1 (HMGB1), secreted ATP, and surface-exposed calreticulin (CRT). It was reported that ferroptosis cancer cells and non-cancer cells could release HMGB1, and then activate co-cultured BMDMs (bone marrow-derived macrophages) to secrete proinflammatory cytokine tumor necrosis factor alpha (TNF-α) via the HMGB1-AGER signaling pathway. This process can be blocked by adding anti-HMGB1 antibodies (101). Therefore, ferroptosis may have the ability to drive macrophage polarization to the M1 type by a HMGB1-mediated immune response. Another study showed that the depletion of *Arntl*, a circadian transcription factor, contributed to increased HMGB1 release and neutrophils recruitment and induced acute pancreatitis, which could be attenuated by anti-HMGB1 neutralizing antibodies and ferroptosis inhibitors (102). This study also suggested that ferroptosis might be involved in HMGB1-induced inflammatory and immune responses, and this thesis was supported by the results of another study (90). Paradoxically, it has been reported that HMGB1 was associated with various cancer progression and poor outcomes (103, 104). However, with there being scarce evidence regarding the impact of ferroptosis-released HMGB1 on tumor growth *in vivo*, it is not enough to conclude that HMGB1 plays a beneficial role in the ferroptosis-induced immune response for antitumor therapy, and further investigations are still required. Calreticulin is another type of DAMPs that has been implicated in eliciting an anticancer effect by initiating a cancer cell death-associated immune response and promoting the increased production of DCs and phagocytotic tumor cells (100). One study has reported the increase of calreticulin exposure on the surface of ferroptotic cancer cells, which, combined with higher emissions of HMGB1 and ATP, induced the activation and maturation of BMDCs to exert an antitumor effect (90). Another important type of DAMPs, ATP, can trigger immunogenic signaling after cancer cell death; and was used as a candidiate for an effective anticancer vaccine (105). In the ferroptosis microenvironment, ferroptosis not only causes mitochondrial fragmentation and reduces ATP levels in neuronal cell (106), but elicits ATP release from ferroptosis cancer cells, and triggers an antitumor immune response (90). Furthermore, in the context of kidney cell death, increasing evidence shows that ferroptosis plays a crucial role in renal cell death, and that lipid peroxides are unique types of ferroptosis-released DAMPs and predominate immunogenic effects (107). Arachidonic acid (AA) peroxidation products and other lipid mediators, including PGE_2_, PE-AA-OOH, oxPLs, and lyso-phospholipids, are emitted from ferroptosis cells. There is no direct evidence that shows that these lipid mediators released by ferroptosis cells have an effect on antitumor immunity in the TME. However, indirect evidence has demonstrated that ALOX12/15-produced phospholipid oxidation products limits the maturation of DCs and the differentiation of fine-tuning T helper 17 (TH17) cells (108). On the other hand, oxidative lipids-equipped lipid bodies are absorbed by DCs and dampen its antigen cross-presentation function and silent subsequent CD8^+^ T cells response in the TME (109). The effect of oxidative lipid mediators on immune response may vary among different disease conditions, cell lines, and animal models. In a word, although there is little direct evidence to support this, ferroptosis-released DAMPs and their associated immune reactions are the potential target to achieve optimal antitumor immunity effect and need to be analyzed further in the future.

**Figure 3:**
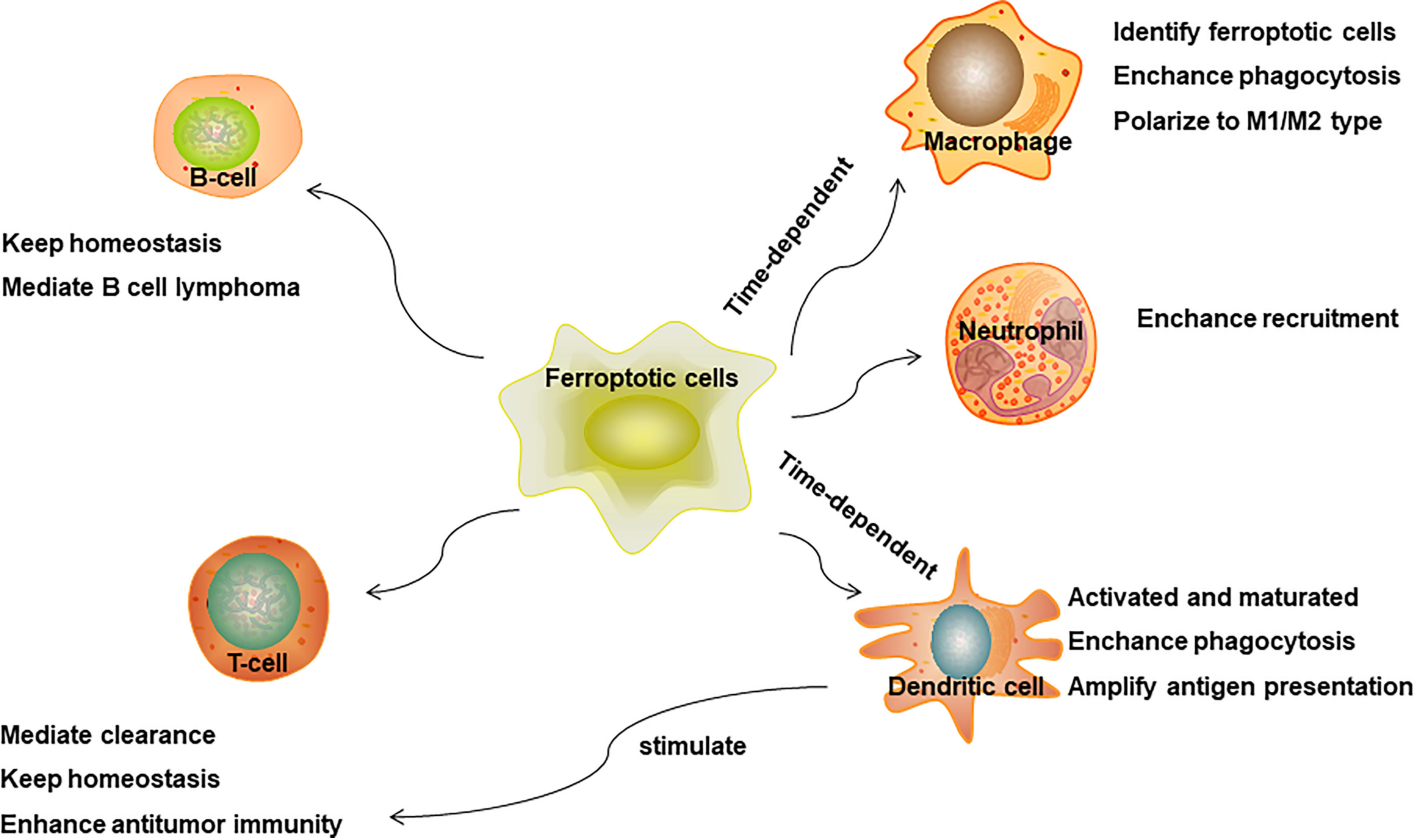
Ferroptosis mediated immune response.

**Figure 4:**
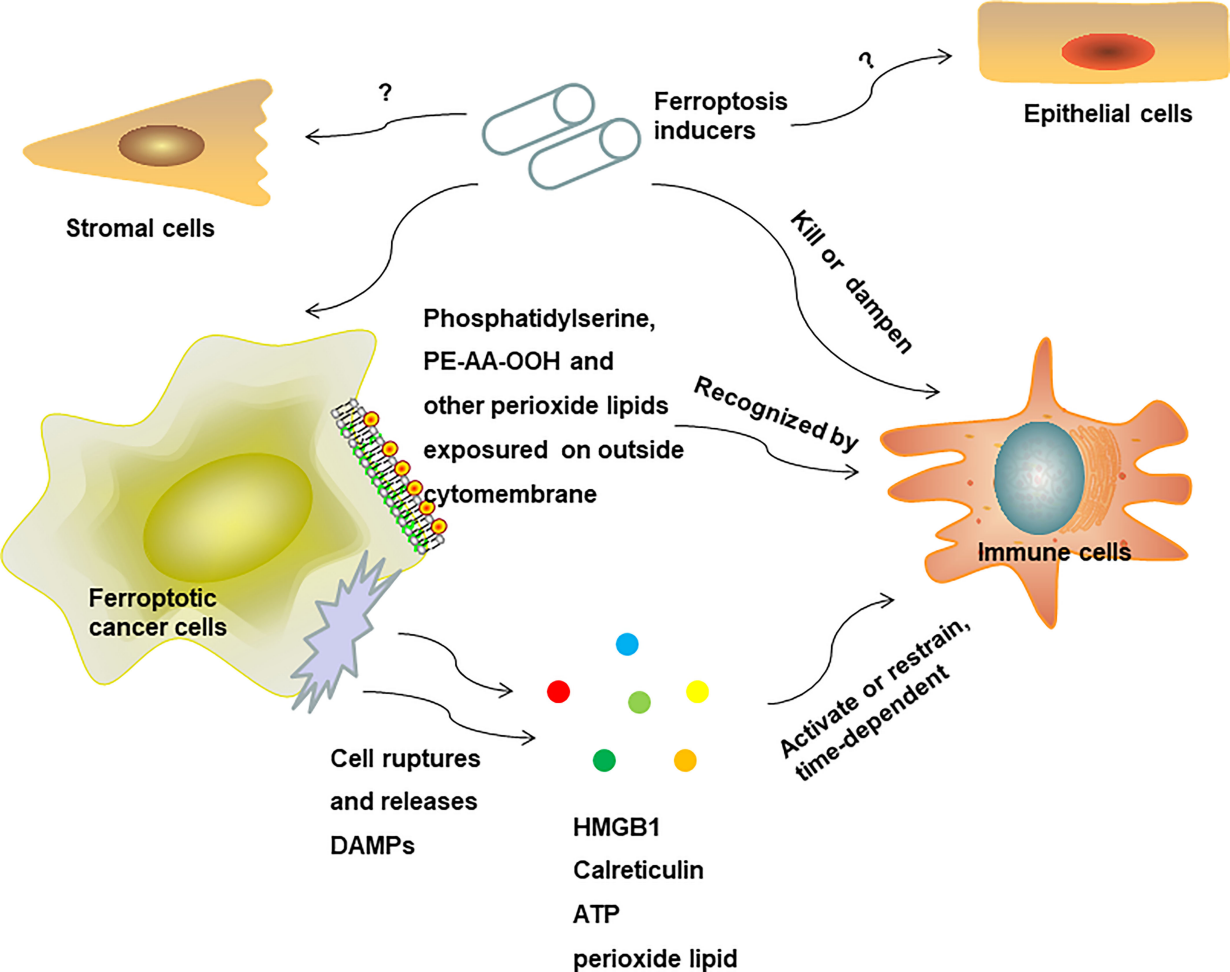
Potential mechanisms for ferroptosis mediated immune response in the TME.

In conclusion, ferroptosis may have an effect on innate immunity reprogramming to favor pro-inflammatory and antitumor response (**Figure 3**), and rely on cell membrane lipid metabolism reprogramming on the surface of ferroptosis cells or releasing soluble antigens as the initial signals to activate innate immune cells (**Figure 4**). Due to a shortage of comprehensive studies and evidence, more studies are required to further explore the impact of ferroptosis on innate immune cells’ reprogramming in diverse disease conditions and TME in the future.

### 3.2 Ferroptosis-associated adaptive immune response

Having explored adaptive immunity responses to the activation of innate immune cells, we move forward to explore the influence of ferroptosis on adaptive immunity. The activation of adaptive immune system by innate immune cells depends on the first and second signals (66). MHC I or MHC II interaction with T-cell receptors is the first signal. DCs then activate and prime T cells, with the second signal as co-stimulator, which predetermines the eventual immune effect cooperating with the first signal. The second signals are often chemokines like IL-1 released by APCs to stimulate T-cell activity (110). Only the first signal without the second signal leads to T cell anergy and immune tolerance (66). How ferroptosis cells prime APCs and further activate adaptive immune cells, and what the first signal and the second signal are during this process, remains obscure. Recently, several studies have focused on ferroptosis associated adaptive immune response. One study showed that ferroptosis was a kind of ICD and promoted maturation of BMDCs, then continuously activated adaptive immune system and inhibited tumor growth *in vivo* (90). Ferroptosis can activate resting memory CD4^+^ T cells and M0 macrophages for igniting anticancer immune response via ferroptosis-related lncRNA in hepatocellular carcinoma (111). Later, but still in the early stages of ferroptosis, ferroptosis cells generate ATP and HMGB1 as immunogenic signals to induce the adaptive immune system and maturation of BMDCs *in vitro* and *in vivo* (112). The time-dependent immunogenicity may be because of early-dying cells being more immunogenic than late-dying cells, meaning that they tend to possess more intact membranes and replicate their intracellular material before it leaks out. In another recent study, ferroptosis cancer cells are divided into three phases in the process: “initial”, related to lipid peroxidation; “intermediate”, associated with ATP release; and “terminal”, defined as a loss of plasma membrane integrity and inceased release of HMGB1s. “Initial” ferroptotic cells reduce the maturation of DCs and impede antigen cross-presentation (113). The contradictory results may arise from specific experimental condition in each study, but whether ferroptosis cancer cells have time-dependent immunogenicity and can be used in vaccines for antitumor immunity still needs to be clarified in studies carried out in the near future.

However, ferroptosis also exerts a direct impact on T cells themselves. One study demonstrated that T-cell clearance was mediated by lipid peroxidation and ferroptosis and that ferroptosis was the vital backup scavenger system that maintained T-cell homeostasis (114). With regard to T-cell immunity, another study demonstrated that T cell-specific GPX4 deficient mice had defective CD8^+^ T cell homeostasis in the periphery and a compromised defense ability against viral and parasitic infection. In *ex vivo* GPX4 deficient T cells accumulated membrane lipid peroxides rapidly and undergone ferroptosis (115). Another study showed that GPX4^-^deficient CD8^+^ T cells absorbed oxidized lipids by the receptor CD36 in the TME were susceptible to ferroptosis and lost their antitumor immunity functionality (116). Furthermore, ferroptosis is also involved in antitumor immunotherapy. The combination of the ferroptosis inducer cyst(e)inase and immune checkpoint inhibitor PD-L1 synergistically facilitates T cell-regulated antitumor immunity, induces ferroptosis, and inhibits tumor growth *in vivo* (22). This work sheds light on the critical role of ferroptosis in immunotherapy in killing tumor cells. Therefore, ferroptosis exerts an effect on T-cell immunity by maintaining the homeostatic balance of T cells, activating the secondary immune response of T cells, and enhancing T cell-mediated anti-tumor immunity. In addition, the deletion of GPX4 in regulatory T cells (Tregs)-facilitated ferroptosis that can enhance the antitumor immune response without inducing overt autoimmunity (117). The other important members in the adaptive immune system are B cells. GPX4 was also found to have the crucial function of maintaining B1 and marginal zone (MZ) B-cell homeostasis, but not that for follicular (Fo) B2 cells (118). The regulation of redox systems plays an essential role in carcinogenesis and cancer development. A clinical and experimental study showed that patients with GPX4-positive diffuse large B-cell lymphoma had significantly poor progression-free survival and overall survival compared with those of the GPX4-negative group by immunohistochemistry results (119). This result might be due to GPX4-positive cancer cells having a powerful reducing capacity that makes them impervious to the effect of ferroptosis triggered by oxidative stress. Hence, targeting GPX4 function, or cysteine levels, or other redox factors may have significant application value for B-cell leukemia treatment. GPX4, a selenium-containing enzyme, is pivotal to regulating the ferroptosis pathway, and the underpinning rationale is that GPX4 is the only enzyme to reduce phospholipid hydroperoxides (49). The depletion of GPX4 renders cancer cells vulnerable to ferroptosis (15) and the therapy-resistant state of cancer cells is dependent on GPX4 being able to escape multiple therapy challenges (20, 21). In addition, GPX4 plays an important role in mediating immune cells themselves function. GPX4 deficient CD8^+^ and CD4^+^T cells fail to withstand virus and parasite infections (115), and in the chicken bursa of the Fabricius model, selenium deprivation elicits a decrease in GPX4, the downregulation of IL-2, IL-6, IL-8, IL-10, IL17, IL-1β, IFN-α, IFN-β, and IFN-γ compared with the controls, and in the bursa having a higher TNF-α level than the controls (120). GPX4 is a potential target that can reshape the TME; its use is thus beneficial in cancer therapy and points to a direction future research can take. System xc– is also crucial to the regulation of ferroptosis and reshaping of the TME. The function of system xc^–^ is to transport cystine into cells at the expense of exporting glutamate out to fulfill cells’ requirement of maintaining a reducing state. Tumor cells utilize this mechanism to evade oxidative stress and ferroptosis. Meanwhile, the exported glutamate can also exert an effect on immune cells in the TME. It was reported that elevated extracellular glutamate contributed to Tregs’ proliferation and activation, thus enhancing immunosuppression in the TME of glioblastoma (121). Another study showed that the loss of system xc^–^ enhanced the anticancer capacity of the immunotherapeutic agent anti-CTLA-4 (122). GPX4 and system Xc^-^ are critical regulators of intracellular metabolic pathways and help to maintain a reducing-state homeostasis. As increasing evidence shows that metabolic environment modification builds up the bridge for crosstalk between cancer cells and immune cells, exploring the role of metabolic reprogramming involved in ferroptosis by targeting GPX4 and system xc^–^ in both tumor cells and immune cells in the TME is a very promising direction for future research.

In conclusion, ferroptosis is the primary mechanism that maintains adaptive immune cells’ homeostasis. However, the overwhelmingly reducing capacity of immune cells may facilitate their development into B cells, or the progressionof leukemia T-cells. More work should be done to clarify how to maintain the proper and delicate balance of redox levels in tumor and immune cells in the TME, and also the optimal way to utilize ferroptosis to inhibit cancer progression in a specific context in the future. GPX4 and system xc^–^ are not only critical regulators of ferroptosis, but also exert a crucial effect on the TME, making them potential targets for cancer therapy.

## 4 Novel applications for ferroptosis-mediated immune response in cancer therapy

In the following text we introduce some new applications for ferroptosis-associated immune response in cancer treatment. In this way, we analyze the close relationship between ferroptosis and immune system and present promising combination therapy strategies that could cure cancer in the near future.

### 4.1 Package ferroptosis inducers into nanomaterials brings antitumor immune response

As certain ferroptosis inducers have short half-lives and potentially systematic adverse effects, the approach of combining ferroptosis inducers with nanomaterials has been intensively implicated in strengthening anticancer immunity in recent years. Zanganeh found that iron oxide nanoparticles (SPIONs) limited tumor growth by regulating macrophages to polarize to pro-inflammatory M1 types in tumor niches (123). Although ferroptosis was not mentioned in this study, abundant iron input probably elevated the labile iron pool burden and elicited the Fenton reaction that leads to ferroptosis. Moreover, SPIONs could directly promote macrophages to undergo classical autophagy and stimulate the expression of inflammatory cytokines at higher levels through the activation of TLR4 signaling via the TLR4-p38-Nrf2-p62 signal axis (124). Furthermore, iron chelated melanin-like nanoparticles (Fe@PDA-PEG) were shown to induce the M2-to-M1 repolarization of tumor-associated macrophages (TAMs), and, in combination with photothermal therapy, synergistically inhibited tumor growth (PTT) (125). The reason for this was that the amplified antigens, stimulated by ferroptosis, acquired the ability of macrophages, and exerted a synergistic effect with the release of PTTinduced tumor-associated antigens (TAAS). Another study demonstrated that αMSH-PEG-C′ nanoparticles could induce tumors to ferroptosis *in vivo* by accelerating iron absorption into cancer cells (126). However, an immunodeficient animal model was used in the study and the ferroptosis-related immune response was not explored. In addition, the engineering magnetosomes, constructed with the core of Fe_3_O_4_ magnetic nanocluster, the cloak of leukocyte membranes with inner face implanted with TGF-β inhibitor and outer face loaded with PD-1 antibody, showed optimal antitumor therapy capacity (127). Biomimetic magnetic nanoparticles Fe_3_O_4_-SAS @ PLT can induce ferroptosis and sensitize the activity of PD-1 immune checkpoint blockade treatment (128). The nanoplatform combining sorafenib (ferroptosis promoter) with photosensitized hemoglobin can strengthen ferroptosis and stimulate an antitumor immune response by recruiting immune cells to secrete IFN-γ (129). There have been plenty of studies conducted in recent years that investigate the activity of nanocomposites, comprising ferroptosis inducers and other components that exhibit amplifying anticancer immunity (130–134). Thus, the synergism of ferroptosis and immunomodulation gives rise to potent therapeutic effects, and exhibits more advantages by uniting characteristics of modified nanomaterial, which is a promising approach for strengthening antitumor immunity for cancer therapy in the future (135).

### 4.2 Ferroptosis-involving combination therapy generates antitumor immunity

Last but not least, we focus on whether ferroptosis-involving combined therapy can induce antitumor immunity. Zou found that uniting reagents that induce ferroptosis with immune checkpoint inhibitors displayed the optimal therapeutic effect for suppressing tumor progression (22). This research also showed that anti-PD-L1 immunotherapy could elicit tumor cell ferroptosis via the CD8^+^ T cell-IFN-γ-system xc^–^ signal pathway. Another study also confirmed the superior synergy of combining ferroptosis with immunotherapy (122, 127). In addition, ferroptosis was found to create bond of synergy between radiotherapy and immunotherapy (23). In addition, the Krebs cycle enzyme fumarate hydratase deprivation (*FH^-/-^*) demonstrated synergy with ferroptosis inducers for the limiting of hereditary leiomyomatosis and renal cell cancer (HLRCC) progression (136). Nanocomposite containing Fe3+ with cancer cell membrane was absorbed by breast cancer cells and induced ferroptosis that could reinforce the synergistic therapeutic effect of sequential PDT and chemotherapy (137). The chemotherapeutics drugs sorafenib was also shown to induce ferroptosis via a ferritinophagy cascade, thus triggering an antitumor immune response (138). In addition, incorporating ferroptosis and ultrasound-triggered sonodynamic therapy (SDT) synergistically elicited strong antitumor immunity by increasing the numbers of mature DCs and activated CD8^+^ cells and decreasing the number of MDSCs in the TME (139). These findings indicate that ferroptosis is capable of synergistic action with chemotherapy, PDT, or SDT, in turn meaning that it can serve as the critical therapeutic target to enhance chemotherapy and PDT treatment outcomes. The underlying synergistic effect mechanism for ferroptosis and chemotherapy may be owing to th ability of these two therapy methods to greatly increase the ROS level in the TME (140). PDT directly leads to ferroptosis in tumor cells, and this would explain the cooperative effect between ferroptosis and PDT (90, 141). We believe that the more intricate and core mechanism of the synergy between ferroptosis and other treatment manners for cancer lies in the potential capacity of ferroptosis to induce a strong antitumor immune reaction, which would augment the therapeutic effect of other treatment methods. In depth, ferroptosis would change tumor cells into “altered self” or “missing self” with the downregulation of the “do not eat me signals” such as CD47 on the surfaces of tumor cells, or through mimicking microbial antigens by releasing DAMPs like oxidized lipids. All these transformations can be recognized by innate immune cells and lead to anticancer immune responses, and these potential mechanisms could be explored and verified in the future. Thus, as the role of ferroptosis in multiple metabolic pathways and in classical cancer treatment approaches of immunotherapy, radiotherapy, chemotherapy, PDT and SDT, has been extensively and thoroughly covered, moving to develop ferroptosis-involving combination therapies for antitumor treatment in the near future is promising (142).

## 5 Conclusion

This review focuses on ferroptosis molecular network and associated immune response in tumor immune niche, but there are some undeniable challenge associated with inducing ferroptosis in the TME. As shown above, although ferroptosis may exert antitumor capacity by killing cancer cells and boosting the immune response, it also has a direct impact on immune cells. In the TME, ferroptosis may also kill immune cells, and disarm the defense force monitoring cancer cells. In addition, normal cells such as vascular endothelial cells and stromal cells would be affected by ferroptosis inducers, which may facilitate metastasis, and the likelihood of this needs further study and confirmation. Therefore, as ferroptosis has a comprehensive influence on all units in the TME, it is crucial to clarify the unique effect on each unit and grasp its overall effect on various cancer types (**Figure 4**). By utilizing novel biological materials and through other physical and chemical means, we could amplify the beneficial role of ferroptosis in cancer therapy and eradicate its potential adverse effect on immune cells and other normal cells in the meantime.

In conclusion, although there is a great number of gaps and dilemmas in this area that needs to be addressed before clinical applications are feasible, we have confidence that working on this subject will lead to a promising future, wherein cancer could be eliminated by the reshaping of tumor immune niches via ferroptosis.

## Author contributions

DQ performed the majority of the research and wrote the manuscript, MP checked and revised the manuscript. All authors contributed to the article and approved the submitted version.

## Glossary

**Table:**

PCD	Programmed cell death
GSDMD	Gasdermin D
TfR1	Transferrin receptor gene
FTH1	Ferritin Heavy Chain 1 gene
FTL	Ferritin Light Chain
VDAC3	Voltage-dependent anion channel 3 (VDAC3)
ATP	Adenosine triphosphate
ETC	Mitochondrial electron transport chain
ROS	Reactive oxygen species
IREB2	Iron response element binding protein
RPL8	Ribosomal protein L8
CS	Citrate synthase
ATP5G3	ATP synthase F0 complex subunit C3
ACSF2	Acyl-CoA synthetase family member 2
TTC35	tetratricopeptide repeat domain 35
GPX4	glutathione peroxidases 4
FSP1	Ferroptosis suppressor protein 1
IFN-γ	Interferon gamma
NFS1	Iron–sulfur cluster biosynthetic enzyme NFS1
HO-1	Heme oxygenase-1
NRF2	Erythroid 2-related factor 2
HCCs	Hepatocellular carcinoma cells
FANCD2	Fanconi anemia complementation group D2
DMT1	Divalent metal transporter 1
PCBP1	poly (rC)–binding protein 1
PHKG2	Phosphorylase kinase G2
ALOXs	Arachidonate lipoxygenases
PUFAs	Polyunsaturated fatty acids
ACSL4	Acyl-CoA synthetase long chain family member 4
LPCAT	Lyso-phosphatidylcholine
PEs	phosphatidylethanolamines
PUFA-ePL	Polyunsaturated ether
GSH	Reduced glutathione
SAPE-OOH	1-steaoryl-2–15-HpETE-sn-glycero-3-phosphatidylethanolamine
AIF	Apoptosis-inducing factor
TGF-β1	Transforming growth factor-β1
GCL	Glutamate-cysteine ligase
GSS	glutathione synthetase
NADPH	Nicotinamide Adenine Dinucleotide Phosphate
TXNRD	Thioredoxin reductase
TCA	Tricarboxylic acid cycle
CDI	Cysteine-deprivation-induced
OXPHOS	Oxidative phosphorylation
α-KG	α-ketoglutaric acid
GOT1	Glutamic-oxaloacetic transaminase 1
FH	Fumarate hydratase
DDR	DNA damage response pathway
HLRCC	*FH-*inactivated renal cell carcinoma
TME	Tumor microenvironment
IKE	imidazole ketone erastin
MHC-I	Class I major histocompatibility complex molecules
PRRs	Pattern recognition receptors
PAMPs	Pathogen-associated molecular patterns
DAMPs	Damage-associated molecular patterns
APC	Antigen presenting cells
PARC	Pathogen response-like chemokine signature
ICD	Immunogenic cell death
PDAC	Pancreatic ductal adenocarcinoma cells
iNOS	Inducible nitric oxide synthase
PS-PDT	Photosensor photodynamic therapy
BMDCs	Bone-marrow derived dendritic cells
MZ	marginal zone
PE-AA-OOH	Phosphatidylethanolamine
PGE2	Prostaglandin E2
oxPLs	Oxidized phospholipids
5-HETE	5 Hydroxyeicosatetraenoic
MDSCs	Granulocytic myeloid derived suppressive cells
HMGB1	High mobility group protein B1
CRT	surface-exposed calreticulin
AA	Arachidonic acid
SPIONs	Iron oxide nanoparticles
Fe@PDA-PEG	Iron chelated melanin-like nanoparticles
TAMs	Tumor associated macrophages
TAAs	Tumor-associated antigens
SDT	ultrasound-triggered sonodynamic therapy.
